# The impact of COVID-19 pandemic on orthodontic services and trainees’ mental health in India

**DOI:** 10.3389/fmed.2023.1220505

**Published:** 2023-09-01

**Authors:** Badri Thiruvenkatachari, Prema Sivakumar, Sanjana Ananth, Yana Sabbagh, Benjamin R. K. Lewis, Stephen M. Chadwick, Gnana Shanmugam Kaliyaperumal, Panchali Batra

**Affiliations:** ^1^School of Dentistry, University of Manchester, Manchester, United Kingdom; ^2^Department of Orthodontics, Sree Balaji Dental College and Hospital, Bharath Institute of Higher Education and Research, Chennai, India; ^3^Betsi Cadwaladr University Health Board, Wales, United Kingdom; ^4^Department of Orthodontics, Countess of Chester Hospital, Chester, United Kingdom; ^5^Department of Orthodontics, Jamia Millia Islamia, New Delhi, India

**Keywords:** COVID-19, orthodontics, questionnaire, recovery survey, trainee mental health

## Abstract

**Aim:**

To evaluate the impact of COVID-19 on clinical orthodontic services, orthodontic education, and the emotional well-being of orthodontists and orthodontic trainees in India.

**Materials and methods:**

The survey was designed using Survey Monkey™ and distributed to members of the Indian Orthodontic Society via their registered email address and also via social media platforms (WhatsApp and LinkedIn).

**Results:**

A total of 610 responses to the survey were received. The majority of respondents agreed on the negative impact of COVID-19 on clinical activity and the associated income of orthodontists. Respondents reported that this was mainly due to national restrictions (70.1%), increased cross infection measures (59.6%), state restrictions (55.9%), and social distancing (39.4%). Ninety one percent of respondents agreed that orthodontic staff should have evidence of vaccination before providing care.

COVID-19 was found to have a negative impact on the trainees’ perceptions of their clinical dexterity (72.4%), their confidence with respect to academic knowledge (66.5%), their mental health (80.7%), and their stress levels during the pandemic (93.2%).

**Conclusion:**

The COVID-19 pandemic has had a negative impact on orthodontic specialists and post-graduate trainees in India. The impact on trainees’ mental health was significantly higher compared to trainees from other countries. Decreased clinical activity has reduced the opportunities for learning, and trainers must rise to the challenge of providing additional support to this cohort of trainees who will progress to become the future orthodontic workforce.

## Introduction

The COVID-19 pandemic began with the identification of a novel coronavirus in the Wuhan province of China in 2019, with the first case identified in India in May of 2020 (1). The virus has caused an unparalleled international crisis, both medically and economically. This has led to an international collaboration to identify new strains of the virus, produce and distribute vaccines, and understand how medical and dental education and specialist care can adapt in the context of our increasing understanding of this new challenge.

The landscape of orthodontic practice as a whole underwent a great deal of modification and change during the COVID-19 pandemic. The pandemic had affected every component and every stage of orthodontic treatment, and the orthodontists were quick to recover and come up with strategies to keep their practice going as best as was possible. Adaptations to the pandemic have included avoiding or minimizing aerosol generation in order to reduce the risk of viral spread and cross-infection. Other adaptations have included the development of remote systems, such as telephone consultations, to reduce face-to-face contacts and travel to clinics in an effort to decrease the risk of exposure for all individuals. Within educational practice, to enable the continuation of teaching and learning, there has been a significant increase in the use of digital technologies, included online learning and video conferencing. However, unlike other courses, the practical nature of orthodontic clinical teaching requires face-to-face contact with patients and teachers, as learning occurs at the same time as clinical care is provided.

During the initial stages of COVID-19, due to the lockdown and stringent national and state guidelines, starting new treatments or cases was challenging and almost non-existent. The most prevalent adjustment during this phase was tele-consultations and online management of emergencies. Orthodontists developed a variety of different strategies for their patients to manage orthodontic emergencies at home and troubleshoot problems which arose (2). Following the acute phase, there was a period of less stringent guidelines where clinics were allowed to function with modified policies, and strict COVID protocols in place (3). Temperature checks, vaccination records, PPE for the doctors and the patients, and spacing out the appointments in order to have fewer patients waiting at the reception area were some of the protocols that were introduced by orthodontists. In India, where most practices are private, the pandemic has caused a huge financial burden for principal dentists (4).

COVID-19 has been difficult for students in training. Although academic groups and study clubs started to take shape and provide a semblance of normalcy for the students (5), the days of clinical practice lost to COVID-19 could not be compensated. This affected their confidence levels and caused mental health deterioration (6, 7).

There are no studies looking at the impact of the COVID-19 pandemic on orthodontic clinics and trainees’ mental health in India. Hence, the aim of the current survey was to evaluate the extent of impact of the COVID-19 pandemic on routine clinical care for orthodontists across India. The survey also evaluated the financial and psychological difficulties faced by orthodontists and trainees during the pandemic.

## Materials and methods

This was a cross sectional questionnaire-based survey from a convenience based sample selected from members of the Indian Orthodontic Society. The study protocol was approved by the Institutional Ethics Committee. Electronic questionnaires were generated using Survey Monkey and included an introduction highlighting the purpose of the survey, the intended publication of its anonymized results and the voluntary nature of participating along with a statement that proceeding with the survey indicated consent to participate. A sample size calculation for 95% confidence interval and 5% alpha and an estimated population of 10,000 orthodontists showed a sample of 370 participants were required. A snowball sampling method was used to recruit participants. The survey was distributed to members of the Indian Orthodontic Society via their registered email address and also by the two most popular forms of social media utilized among the profession (WhatsApp and LinkedIn). The Heads of Orthodontic Departments of academic institutes were contacted requesting to circulate the questionnaire to their trainees and academic staff. The authors helped in circulating the questionnaire within their professional network.

A 35-item questionnaire (Supplementary data) was developed based on a previously published survey on the COVID-19 experience of orthodontists in the United Kingdom (8). However, the questions utilized were adapted for an India orthodontic workforce. Additionally, a separate section was included on the opinion of orthodontic trainees on their clinical education; academic education; and any mental health problems they had experienced during the pandemic. The questionnaire included five domains:

1. Demographic data.
2. Pandemic and dentistry.
3. Effects on the delivery of care.
4. Living with COVID-19.
5. Academic and clinical training.

The first domain included demographic information and workplace information using multiple choice and semi open questions. The next domain on orthodontists’ knowledge on the pandemic and dentistry included five Likert-scale items testing their knowledge on the pandemic and its impact on dentistry. The impact on the delivery of care included four Likert-scale questions and two multiple choice questions on their knowledge and behaviors. The domain on living with covid included 10 Likert-scale questions and one multiple choice question on their expected behaviors after resumption of services post covid pandemic. The dedicated trainee section had one multiple choice question on their year of training and seven Likert-scale questions on the impact of the covid pandemic on their academic activities and their mental health status.

The questionnaire survey responses were collected through Survey Monkey. The questionnaire link could be opened only once to restrict multiple responses from participants. The participants were provided with a contact number from the coordinating team for assistance with any issues they encounter during survey completion. However, the team was made aware that only IT issues were to be assisted and no other information on questionnaire/questions will be provided. Each participant had 4 weeks to complete the survey and a reminder was sent after 2 weeks for those who failed to respond.

### Statistics

The responses from the Survey were downloaded from the online survey platform. For open ended and semi open-ended questions, the responses were transferred to excel spread sheet independently and in duplicate by two authors (SA and PS). Any discrepancies were resolved on discussion with the third author (BT).

Descriptive statistics were performed for all categories and presented as numbers and percentages. Descriptive texts from open ended questions were segregated by two authors independently and in duplicate. The answers were reported descriptively as texts.

## Results

### Demographics

Of the 610 responses, 54 were completed by general dentists/non orthodontists and 556 were either specialists or training orthodontists. The overall number of male respondents (51.1%) was almost equal to the number of female respondents (48.7%). However, there were significant variations between the various age groups. 60.8% of respondents were under 35 years of age and within this cohort 61.1% of the respondents were female with 38.6% being male. There was an opposite gender split in the over 35 s with 29.5% being female and 70.5% being male with a total of 39.2% (Supplementary Table S1).

Around one third of the respondents identified themselves as orthodontists in training (36.6%), 29.5% as academic orthodontists and 34% as qualified orthodontic specialists (Supplementary Table S2). The majority of our respondents worked in tertiary care (57.6%). Around 43.9% worked in primary care practice setting and 19.9% worked in a secondary care setting (private and public hospital). Interestingly, 12.1% worked in highstreet general dental practice setting (Supplementary Table S3a). Based on locations of practice, around 130 respondents worked in multiple sites with 2.4 locations per person. Around 75% practiced in a single setup (Supplementary Table S3b).

### Pandemic and dentistry

Ninety-five percent of our respondents agreed that COVID-19 had had a significant impact on the clinical service provision and 92.4% agreed on the negative impact on revenue generation of orthodontic practitioners. Respondents felt that dentistry (16.1%) and orthodontics (18.2%) were equally well prepared for a pandemic like COVID-19. Nearly half of the respondents (46.3%) agreed that orthodontics services have coped well during the pandemic and the majority felt that COVID-19 would be an ongoing issue (95%) (Table 1).

**Table 1:** Responses to questions on pandemic and dentistry.

Questions	Strongly agree *N* (%)	Agree *N* (%)	Neutral *N* (%)	Disagree *N* (%)	Strongly disagree *N* (%)
Dentistry was prepared for a pandemic like COVID-19	12 (2.3%)	71 (13.8%)	99 (19.2%)	235 (45.5%)	99 (19.2%)
Orthodontic services were prepared for a pandemic like COVID-19	15 (2.9%)	79 (15.3%)	101 (19.6%)	243 (47.1%)	78 (15.1%)
Orthodontic services have coped well during the COVID-19 Pandemic	29 (5.6%)	210 (40.7%)	128 (24.8%)	132 (25.6%)	17 (3.3)
COVID-19 will be an ongoing issue for all clinical services	199 (38.6%)	291 (56.4%)	15 (2.9%)	10 (1.9%)	1 (0.2%)
COVID-19 will be an ongoing issue for our society	227 (44.0%)	269 (52.1%)	12 (2.3%)	7 (1.4%)	1 (0.2%)
COVID-19 has affected our ability to see and assess new orthodontic patients	115 (22.8%)	271 (53.8%)	48 (9.5%)	61 (12.1%)	9 (1.8%)
COVID-19 has affected our ability to see patients in active treatment	107 (21.2%)	301 (59.7%)	41 (8.1%)	52 (10.3%)	3 (0.6%)
COVID-19 has significantly affected clinical revenue with respect to orthodontic practitioners	178 (35.3%)	288 (57.1%)	29 (5.8%)	7 (1.4%)	2 (0.4%)

More than two thirds of our respondents agreed that COVID-19 has affected their ability to see and assess new orthodontic patients (76.6%), and their ability to start new courses of treatment (80.3%) (Table 1).

The most frequently reported adaptation to practice has been the use of telephone consultations (63.6%) followed by video (47%), extended opening times (19.1%) and hiring more staff (5.5%) and other measures (11.2%). Interestingly, a small number of respondents took no additional measures (9.8%) (Table 2). The main influences to changes to clinical practice during the pandemic have been as a result of national restrictions and lockdowns (70.1%), state restrictions (55.9%), Dental Council of India (DCI) guidelines (34.3%), Indian Orthodontic Society (IOS) guidelines (23.6%), increased cross-infection measures (59.6%), sickness or self-isolation of staff (29.9%), shutting down clinic due to monetary issues (15%), patient related monetary issues (30.5%), patients’ fear of attending the clinic (63.6%), social distancing policies (39.4%) and other reasons (0.2%) (Table 3).

**Table 2:** Table describing adaptation in clinical practice to suit pandemic crisis.

Level of agreement	Number	Percentage of respondents
No additional measures	52	9.8
Telephone consultations	336	63.6
Video consultations	248	47.0
Extended opening times	101	19.1
Hired more staff	29	5.5
Other	59	11.2
Total	773	-

**Table 3:** Table describing factors which resulted in changes to clinical practice during the pandemic (508 responses-tick all that apply).

Cause of Interruption	Number	Percentage
National restrictions/lockdown	356	70.1
State restrictions	284	55.9
Dental council of India guidelines	174	34.3
Indian orthodontic society guidelines	120	23.6
Increased cross infection measures	303	59.6
Staff sickness/self-isolation	152	29.9
Clinic shut due to monetary issues	76	15.0
Patient related monetary issues (Job loss/no income)	155	30.5
Patient’s fear of attending clinic	323	63.6
Social distancing policies	200	39.4
Other	1	0.2

On an average, the number of measures taken overall were 1.46 per person, and the average number of measures taken by those who took measures were 1.62 (476 respondents) and the average number of measures taken by those who took more than one measure was 2.13 (263 respondents).

### Living with COVID-19

The majority of respondents agreed that following the COVID-19 pandemic, orthodontic staff should be regularly tested for the virus to reassure patients (71.7%), with a monthly testing regime being the most favored (39.6%) (Table 4), and that there will be a need for continued increased cross infection control measures (90.5%) and difficulty in arranging extractions (63.5%) leading to a delay in orthodontic treatment. Ninety-one percent of respondents agreed orthodontic staff should have evidence of vaccination before providing care. Nearly 78% of respondents agreed that during the pandemic patients’ compliance has suffered, 69.6% respondents identified patients’ oral hygiene had deteriorated, and 58.7% felt that orthodontic patients are more likely to discontinue treatment before achieving the treatment’s aims (Table 4). Interestingly, one quarter of the respondents felt orthodontic services will get back to normal in 6 and 12 months, respectively, (Supplementary Table S4).

**Table 4:** Responses for questions on living with COVID-19.

Questions	Strongly agree *N* (%)	Agree *N* (%)	Neutral *N* (%)	Disagree *N* (%)	Strongly disagree *N* (%)
Following COVID-19, there will be additional cross-infection control	173 (35.5%)	268 (55.0%)	34 (7.0%)	9 (1.8%)	3 (0.6%)
Following COVID-19, there will be delays in orthodontic treatment due to the difficulty in arranging extractions	66 (13.6%)	243 (49.9%)	79 (16.2%)	94 (19.3%)	5 (1.0%)
Following COVID-19 Orthodontic staff should be tested regularly	85 (17.5%)	264 (54.2%)	84 (17.2%)	50 (10.3%)	4 (0.8%)
Following COVID-19, Orthodontic staff should have evidence of vaccination before providing clinical care	202 (41.5%)	241 (49.5%)	30 (6.2%)	10 (2.1%)	4 (0.8%)
Following COVID-19, patients in active orthodontic treatment have had a deterioration in their oral hygiene	82 (16.8%)	257 (52.8%)	105 (21.6%)	41 (8.4%)	2 (0.4%)
Following COVID-19, orthodontic patients are more likely to request to stop treatment before the aims of treatment have been achieved	52 (10.9%)	233 (47.8%)	96 (19.7%)	93 (19.1%)	12 (2.5%)
Following COVID-19, there will be an improvement in our quality (outcomes) of orthodontic care	37 (7.6%)	173 (35.5%)	190 (39.0%)	73 (15.0%)	14 (2.9%)
Following COVID-19 orthodontic patients’ compliance has suffered	78 (16.0%)	301 (61.8%)	71 (14.6%)	35 (7.2%)	2 (0.4%)

Neary 70% of the respondents agreed that COVID-19 pandemic had a negative impact on their mental health. Only 13% of the respondents felt the pandemic as having no impact on their mental health. When the responses were analyzed for trainees, the results showed that 80% had negative impact on mental health and 6% reported as having no impact (Table 5).

**Table 5:** Responses to question ‘COVID-19 has impacted your mental health negatively’.

Level of agreement	Overall		Trainees		Non-Trainees	
	Number	%	Number	%	Number	%
Strongly agree	124	25.5	79	35.4	45	17.0
Agree	216	44.4	101	45.3	115	43.6
Neutral	80	16.2	37	16.6	43	16.3
Disagree	51	10.5	5	2.2	46	17.4
Strongly disagree	16	3.3	1	0.4	15	5.7
Total	487	99.9	223	99.9	264	100

### COVID-19 and impact on trainees

Out of total trainee respondents 31.8% belonged to first year, 23.3% in second and 43.9% in third year (Supplementary Table S5). Ninety-three percent of respondents agreed following COVID-19 there had been a spike in mental stress levels and 69.9% of total respondents agreed that COVID-19 had a negative impact on their mental health with 80.7% being trainees and 60.6% being non-trainees (Table 5).

The trainees also reported a negative impact of COVID-19 on their perception of their clinical dexterity (72.4%) and their confidence with respect to academic knowledge (66.5%). Eighty seven percent of respondents agreed patients had discontinued treatment due to the effects of the pandemic, 94.9% trainees agreed on seeing decreased number of patients per day. When asked about the impact of COVID-19 on their clinical targets, 89.1% of respondents agreed the pandemic will affect their ability to complete their Dental Council of India targets on the number of finished cases and 96.4% faced difficulty in finishing planned treatments and displaying them for their final examination (Table 6).

**Table 6:** Orthodontic trainees’ responses to COID-19 impact on their academic and training.

Questions	Strongly agree *N* (%)	Agree *N* (%)	Neutral *N* (%)	Disagree *N* (%)	Strongly disagree *N* (%)
How much do you agree or disagree with the following statement? The number of patients you see on a regular basis has decreased	117 (60.0%)	68 (34.9%)	6 (3.1%)	4 (2.1%)	0 (0%)
Do you feel that you have lost patients since COVID-19? (Deboned braces/continued treatment elsewhere)	94 (42.5%)	99 (44.8%)	25 (11.3%)	2 (0.9%)	1 (0.5%)
Do you think COVID-19 will affect your ability to complete your DCI Quota target?	125 (56.8%)	71 (32.3%)	13 (5.9%)	10 (4.5%)	1 (0.5%)
Do you think COVID-19 has caused a break in continuity of planned treatment making it difficult to finish treatment for patients you had planned to display for exams?	137 (62.3%)	75 (34.1%)	5 (2.3%)	3 (1.4%)	0 (0%)
Do you feel your clinical dexterity has deteriorated impacting your confidence levels?	67 (30.3%)	93 (42.1%)	32 (14.5%)	23 (10.4%)	6 (2.7%)
Do you feel less confident with respect to your academic knowledge post-pandemic?	53 (24.0%)	94 (42.5%)	40 (18.1%)	27 (12.2%)	7 (3.2%)
Following COVID, do you think there has been a spike in mental stress experienced by students?	97 (44.1%)	108 (49.1%)	12 (5.5%)	2 (0.9%)	1 (0.5%)

The survey posted “Freetext” space to allow for additional comments by the respondents. The majority were regarding the additional measures utilized during the pandemic and the influences to changes in clinical practice. Some of these are provided below:

Responses for the question ‘*How did you adapt your practice to suit the pandemic crisis?*’:

- *Sanitization, increase in patient and instrument cross infection measures, limiting the number of patients seen in day* etc.
- *Only provided emergency treatment*
- *By sticking to disease control measures it allowed us to keep practicing.*
- *Increased time for disinfection after each patient*
- *Seeing one patient at a time, keeping more time between two patients, reduced clinical hours, more measures for protection, disinfection and sanitation.*
- *Extra oral suction, air purifiers, fumigation machine, 3 M face mask, face shield, aprons, thicker gloves, sanitizers and disinfectant*
- *Proper appointment schedule to undertake proper asepsis.*
- *Structural changes in clinic, reduced working hours, increased appointment intervals, use of more disposables*
- *Adapting new protocols in sterilization and sanitation*
- *Consultations only by appointment*
- *Installed more dental chairs, more instruments, more infrastructure*
- *HEPA filters, UV light, surface disinfectant* etc.
- *Anti-virus fumigation, contact less dentistry, massive role of oral prebiotics & probiotics, foot pedal disinfectant stands, use of alkaline water RO machine.*
- *Kept more time between appointments, tried to keep the clinical area well ventilated*
- *Alterations in operating techniques, critical case selection*
- *Reduced patient flow. Avoided private practice for some time*
- *Patients asked to get a negative covid test*
- *Took as much precautions as possible*
- *Adequate precautions and spaced appointments*
- *PPE, ultraviolet sterlization of operatory, fogging etc.*
- *Hepa filters, use of kn 95, more time for environmental cleaning between each patients, 1 day prior telephone triage.*
- *Use of proper ppe with limited number of patients per day*
- *Minimal invasive treatment options*
- *Patients for emergency only*
- *PPE, masks, gloves, automatic hand sanitizer, UV OT, air purifier, etc*
- *Rigorous use of PPE and covid appropriate behavior.*

Responses to the question ‘during the pandemic what were the main causes to changes in your clinical practice’

- *Covid duty and self-quarantine*
- *Indian dental association, department of health regulation guidelines*
- *social distancing not possible due to lack of space*
- *City guidelines, biometric identification* etc.
- *Fear of getting infected*

## Discussion

The present survey showed that the COVID-19 pandemic had a significant impact on orthodontic services across India. More than 90% of respondents felt that it significantly reduced their clinical activity and their revenue, and that the pandemic would be an ongoing issue. The pandemic also had a devastating impact on trainees’ mental health with 93% reporting a spike in mental stress levels and more than two-thirds reporting COVID-19 having a negative impact on their mental health.

The survey had a total of 528 responses. A recent survey by Rakhyani et al., reported on orthodontists’ apprehension regarding COVID-19 and received 314 responses (9). Sabbagh et al., described the COVID-19 experience of orthodontists in the United Kingdom and reported data from 560 unique responses (8). Although the present survey sample was small compared to the size of the Indian orthodontic population, the sample represented good geographic diversity and can be considered a reasonable representation of the target population. Additionally, our sample size was 380 participants.

Over half of our respondents were between 25 and 34 years of age with only 4.9% of respondents over 55 years of age. This is in contrast to the United Kingdom study that reported more respondents who were aged over 50 years than respondents who were aged below 50 years (8). This could be due to a combination of the overall younger demographics of the orthodontic providers within India as well as the distribution methods utilized for this survey. The addition of questions related to orthodontic trainees and their experience during COVID-19 may also have influenced this age distribution.

There was a nearly equal split in respondent gender (270 males to 257 females). However, there were more females than males in the younger age group (<35 years). Although this could have introduced bias in the sample, this is well aligned with the national data of orthodontists registered with the Indian Orthodontic Society, which has more females in the younger orthodontic age group and more males in the older orthodontic age group.

The results for the ‘type of professional’, were different between India and the United Kingdom (8) in their distribution. The Indian survey had more orthodontic trainees than the UK (78.1% as opposed to 21.9%), and the UK had more general dental practitioners with a special interest in orthodontics. This could be attributed to the fact that we circulated the questionnaire only among orthodontists in India.

The results for the preparedness of dentistry and orthodontics for the COVID-19 pandemic were unsurprising and in accordance with the results from previous studies (8, 10). However, nearly half of respondents felt orthodontics coped well during the pandemic. These results are contrary to the results reported from previous studies (8). This could be because of the low number of positive cases in India and shorter lockdown periods during the first wave, thus allowing time for orthodontists to be better prepared for the second wave. More than 90% of the respondents thought COVID-19 would be an ongoing issue, both for clinical services and the society. This is in contrast to a previous study that reported only 70% of respondents agreeing that this to be an ongoing issue (9). This difference may be related to the different timepoints at which the surveys were conducted and the negative outlook that the emergence of variants could generate within the profession.

Nearly all the respondents agreed that COVID-19 has had a negative impact on orthodontic clinical services and associated revenue (92.4%). The situation is not much different from other health care systems in India and globally. It is important to point out that private health care systems, including orthodontic and dental services, received no government funded stimulus packages or staff furlough schemes. A recent survey by the American Association of Orthodontists showed similar results, but they reported that 93% of orthodontists managing financial aspects received government/public funded support during the COVID-19 pandemic (11). Across the globe, revenue generation from orthodontic clinics fell during the COVID-19 pandemic due to a reduction in patient throughput, despite providers increasing their hours to provide improved treatment capacity and to try and compensate for the impacts of the pandemic to a limited extent (4, 12, 13). Cross infection measures, social distancing, Cancellations due to testing positive and the need for self-isolation, increased fail-to-attend rates, fallow times, and the time taken for increased cross-infection measures between patients all had a negative effect on productivity within the clinical environment.

Telephone and video consultations were the two most popular adaptations introduced by the respondents. The respondents felt that following the pandemic, both were likely to continue to become part of the “new normal.” These are not new to clinical care and have been acknowledged prior to the pandemic as a mechanism to provide efficient care by significantly reducing travel time and cost (14). However, as a result of the pandemic and the need to provide advice and care remotely, they have become much more accepted and normalized among both the profession and the public (15). A recent systematic review identified that most orthodontic emergencies can be managed by teleassistance and can be used as a routine tool to reduce unnecessary office visits (16).

When asked about the factors that influenced changes to clinical practice, most respondents reported national restrictions as the main reason, followed by state guidelines and DCI guidelines. It is heartwarming to see that the DCI and the IOS played an important role in helping orthodontists adapt to the COVID-19 pandemic. In a country like India, it is extremely difficult to have one-size-fits-all policies, but the survey points out that national organizations in India, with their guidance and directions, have been of huge assistance for orthodontists at a time of crisis.

It is important to point out that 15% of respondents had to close their practice due to monetary issues. This situation is unique for India, as there is no government support or aid for private health care, although over 87% of services are provided in the private sector (17). Additionally, 30% of respondents reported that patient related monetary issues were one of the reasons for changes in clinical practice. The possible reason could be the lack of furloughs as in the United Kingdom or similar government aided schemes in India during the COVID-19 pandemic.

More than half of the participants agreed that it has been difficult to see and assess new orthodontic patients and start courses of treatment. This could be because of the public restrictions and hesitance to start a course of orthodontic treatment at a time of worldwide health emergencies. The results for whether there will be treatment delays due to difficulty in arranging extractions had a huge variation compared to a previous study (8), and this could be because of the nature of dental practice in India, with comprehensive private clinical practices that have consultants from different specialties working as visiting associate specialists.

Although 91% of respondents agreed that the orthodontic staff should have evidence of vaccination before providing care, some respondents have insisted on adult patients being vaccinated and having a negative COVID-19 report before attending to reduce the risk of cross infection. This demonstrated the perceived need for reassurance, both among the patient group as well as the orthodontic workforce.

### Impact of COVID-19 on orthodontic trainees

The effects of lockdown on the mental health of dental students have been recognized, with high levels of depression, anxiety, and stress reported (6, 7). Orthodontic clinical education requires face-to-face contact with patients, as learning occurs simultaneously as care is provided. Clinical skills, the ability to evaluate treatment progress, patient/professional communication, and technical skills are difficult to both teach and learn when de-contextualized. Although the orthodontic profession has helped to develop online learning through virtual learning environments, these were always designed to support clinical chairside teaching and not as a substitute for it (18). The present survey showed 81% of trainees reporting negative impacts on their mental health. The figures are in accordance with postgraduate dental trainees in Wales, with 90% of them reporting negative effects on their mental health (19). However, that survey included a smaller sample than the present study.

The survey shows the reduced amount of clinical activity performed by postgraduate trainees in India during the COVID-19 pandemic. More than 90% of the trainee respondents reported reduced clinical activity, an inability to complete their targets, and an inability to complete an adequate number of cases for the examination. This highlights their anxiety and its impact on their overall confidence. In response to the pandemic, online assessment has been pioneered (20); however, examinations can evidence competence and certificate understanding, but there is no comprehensive digital compensation that can ameliorate the lack of face-to-face learning opportunities resulting from the pandemic, if you aspire to become a specialist orthodontist. Trainees are cognizant of this lack of exposure to learning in clinic, and this has added to their stress, particularly for those coming up to their final summative assessments. It is paramount that trainers do all they can to help support their trainees, as this survey has highlighted the significant impact that the COVID-19 pandemic has had on post-graduate trainees in India. The time taken for increased cross infection control measures, social distancing, and testing for the virus are all important to reduce the spread of the virus, but all serve to reduce clinical activity and opportunities for learning. Trainees need reassurance that despite the pandemic, they will develop the skills, competencies, and understanding they need to become orthodontists.

### Limitations

The study has three main limitations. The response rate was low compared to the overall Indian orthodontic population. However, the sample had a reasonably wide geographic distribution across India, thereby making the study more generalizable. The second limitation is that the sample included more females in the younger age group and more males in the older age group. This, however, reflects the wider orthodontic population across India, with more female trainees than male trainees registered with the Indian Orthodontic Society. Finally, as this is a questionnaire-based study, there is potential for recall bias with respondents failing to recall previous experiences accurately. Although recall bias is a potential issue, we feel the impact is reduced as the questions were carefully framed to reduce the number of questions requesting historical information.

## Conclusion

The survey showed that the COVID-19 pandemic had a significant impact on orthodontic services across India. More than 90% of respondents felt that it significantly reduced their clinical activity and their revenue, and that the pandemic would be an ongoing issue.

The COVID-19 pandemic has had a negative impact on the mental health of orthodontic specialists and post-graduate trainees in India. Decreased clinical activity has reduced the opportunities for learning, and trainers must respond to support this cohort of trainees, who will eventually progress to become the next generation of orthodontic workforce in India.

## Data availability statement

The original contributions presented in the study are included in the article/Supplementary material, further inquiries can be directed to the corresponding author.

## Author contributions

YS, SC, BL, and BT conceived the idea and developed the questionnaire. PS, SA, GK, PB, and BT distributed the questionnaire and collected data. BT, BL, SC, PS, and SA analyzed the data and prepared the draft. All authors were involved in writing up the manuscript.

## Funding

Although this work was not funded, the corresponding author has funding support from India Alliance DBT/Wellcome Trust, and this project was carried out during this time.

## Conflict of interest

The authors declare that the research was conducted in the absence of any commercial or financial relationships that could be construed as a potential conflict of interest.

## Publisher’s note

All claims expressed in this article are solely those of the authors and do not necessarily represent those of their affiliated organizations, or those of the publisher, the editors and the reviewers. Any product that may be evaluated in this article, or claim that may be made by its manufacturer, is not guaranteed or endorsed by the publisher.
