# Supplementary material for: The impact of COVID-19 pandemic on orthodontic services and trainees’ mental health in India
Source: Front Med (Lausanne). 2023 Sep 1;10:1220505. doi: 10.3389/fmed.2023.1220505 (PMC10505436; doi:10.3389/fmed.2023.1220505)
Supplement: Supplementary file 1 [file Data_Sheet_1.docx]

| Age Range (Years) | Percentage | Total | Male | Female | Prefer not to say |
| --- | --- | --- | --- | --- | --- |
| <18 | 0 | 0 | 0 | 0 | 0 |
| 18-24 | 5.5 | 29 | 7 | 22 | 0 |
| 25-34 | 55.3 | 292 | 117 | 174 | 1 |
| 35-44 | 23.1 | 122 | 82 | 40 | 0 |
| 45-54 | 11.2 | 59 | 47 | 12 | 0 |
| 55-64 | 3.8 | 20 | 12 | 8 | 0 |
| 65+ | 1.1 | 6 | 5 | 1 | 0 |
| Total | 100 | 528 | 270 | 257 | 1 |

Supplementary Table s1: Age and gender of the respondents

| Professional Type | Number | Percentage |
| --- | --- | --- |
| Academic Orthodontist (attached to a college) | 156 | 29.5 |
| Orthodontic specialist | 179 | 33.9 |
| Orthodontist in training | 193 | 36.6 |
| Other | 0 | 0 |
| Total | 528 | 100 |

Supplementary Table s2: Table describing profession of respondents

| Location | Number | Percentage |
| --- | --- | --- |
| General Practice | 64 | 12.1 |
| Private Practice | 232 | 43.9 |
| Training Institute | 304 | 57.6 |
| Government Hospital | 39 | 7.4 |
| Private Hospital | 66 | 12.5 |
| Primary Health Centres | 3 | 0.6% |
| Other | 2 | 0.4% |
| Total | 710 |  |

| Overall 1.34 locations per individual |
| --- |
| Of the 130 who worked in multiple sites, it was an average of 2.4 locations |
| Multiple places: |
| 1 – 398 – 75.4% |
| 2 – 92 – 17.4% |
| 3 – 26 – 4.9% |
| 4 – 11 – 2.1% |
| 5 – 0 – 0% |
| 6 – 1 – 0.2% |

Supplementary Table s3a and s3b: Tables describing location of practice

| Time Period | Number | Percentage |
| --- | --- | --- |
|  |  |  |
| <3/12 | 63 | 12.9 |
| 3-6/12 | 126 | 25.9 |
| 6-9/12 | 90 | 18.5 |
| 9-12/12 | 54 | 11.1 |
| 12/12+ | 139 | 28.5 |
| Never | 15 | 3.1 |
| Total | 487 | 100 |

Supplementary Table s4: Responses to question ‘*Following COVID-19 orthodontic clinical activity will return to pre-COVID levels in*’:

| Year of Training | Number | Percentage |
| --- | --- | --- |
| Year 1 | 71 | 31.8 |
| Year 2 | 52 | 23.3 |
| Year 3 | 98 | 43.9 |
| Unlabelled | 2 | 0.9 |
| Total | 223 | 99.9 |

Supplementary Table s5: Year of training
